# Laboratory and Greenhouse Assessments of *Steinernema Carpocapsae* With Three Adjuvants on *Chrysodeixis Includens*

**DOI:** 10.2478/jofnem-2025-0034

**Published:** 2025-09-17

**Authors:** Minling Zhang, Nathan R. Spaulding, Gadi V.P. Reddy, David I. Shapiro-Ilan

**Affiliations:** Southern Insect Management Research Unit, United States Department of Agriculture Agricultural Research Service, 141 Experiment Station Rd., P.O. Box 346, Stoneville, MS 38776; Southeastern Fruit and Tree Nut Research Station, United States Department of Agriculture Agricultural Research Service, 21 Dunbar Rd., Byron, GA 31008

**Keywords:** adjuvant, *Chrysodeixis includens*, soybean looper, *Steinernema carpocapsae*

## Abstract

*Chrysodeixis includens* (Lepidoptera: Noctuidae) is a significant soybean pest in the southern United States. As it has developed resistance to many commonly used insecticides, alternative control measures are necessary. Entomopathogenic nematodes (EPNs) may be one such alternative. Our previous study found that a surfactant Southern Ag Surfactant (SAg Surfactant), significantly increased the mortality caused by *Steinernema carpocapsae* on the first instars of *Helicoverpa zea* in corn plants. In this study, SAg Surfactant and two more adjuvants — dish soap and vegetable oil — were tested for efficacy of *S. carpocapsae* on *C. includens* larvae under laboratory and greenhouse conditions. The three adjuvant treatments tested were 0.125% dish soap (Soap), dish soap combined with 0.25% vegetable oil (Soap & Oil), and 0.066% SAg Surfactant. In laboratory conditions, insect mortality caused by *S. carpocapsae* 72 hr after application was significantly higher with the Soap & Oil treatment than with the no-adjuvant treatment at 1 and 4 hr of exposure, as well as with the Soap treatment at 4 hr of exposure. No significant difference was observed among the EPN with and without adjuvant treatments when the exposure times were extended to 8 and 24 hr. However, compared to the no-adjuvant treatment, insect mortality 24 hr after application was significantly higher for all EPN adjuvant treatments at 8 hr of exposure and for the Soap & Oil treatment at 4 and 24 hr of exposure. These results suggest that these adjuvants shortened the time needed for EPNs to kill *C. includens* larvae. In the first trial, under greenhouse conditions, insect mortality 72 hr after application was not affected by the adjuvant treatments. In the second trial, all the adjuvant treatments increased insect mortality. However, in the third trial, only the Soap & Oil treatment caused higher mortality compared to the no-adjuvant treatment. Additionally, the Soap & Oil treatment yielded the highest number of viable EPNs in most of the three trials, although this result was statistically significant only at one sampling point. Overall, our results showed that the adjuvants could enhance the efficacy of *S. carpocapsae* on *C. includens* larvae.

*Chrysodeixis includens* (Walker) (Lepidoptera: Noctuidae), commonly referred to as soybean looper, is an economically significant crop pest in the southern United States (Debnath et al., 2024). It causes damage to vegetable and field crops, including soybean, cotton, alfalfa, sweet potato, and clover (Hensley et al., 1964). Due to the frequent use of pesticides in its host crops, *C. includens* has developed a resistance to many commonly-applied insecticides (Felland et al., 1990; Thomas and Boethel, 1994; Mascarenhas and Boethel, 1997). While current varieties of transgenic soybeans remain protected against *C. includens*, evidence indicating more widespread Bt crystalline (cry) protein resistance among various insect species is a cause for concern (Tabashnik and Carrière, 2017; Horikoshi et al., 2021). Therefore, there is a need to explore alternative control measures.

Entomopathogenic nematodes (EPNs) are an alternative to chemical pesticides for controlling *C. includens*. However, the efficacy of soil-dwelling EPNs against foliar insect pests is limited by factors such as desiccation and UV radiation (Gaugler and Boush, 1978; Gaugler et al., 1992; Glazer, 1992). Selecting EPN strains that are tolerant of these conditions could facilitate their foliar use. *Steinernema carpocapsae* (Weiser) has previously been shown to have desiccation and UV radiation tolerance, making it a promising candidate for foliar applications (Patel et al., 1997; Shapiro-Ilan et al., 2015). Along with the selecting strains that are tolerant of desiccation and UV radiation, studies have also shown that adjuvants can aid in the control of foliar pests with EPNs (Kaya et al., 1981; Glazer et al., 1992; Baur et al., 1997; Mason et al., 1998; Schroer and Ehlers, 2005; Schroer et al., 2005a, b; Navaneethan et al., 2010; Beck et al., 2013; Portman et al., 2016; Zhang et al., 2024). Schroer et al. (2005a) categorized potential enhancers into three functional groups: surfactants (wetting agents or detergents), binders/thickeners (polymers), and oil/wax. They evaluated a range of surfactants, polymers, and combinations of the two, emphasizing that surfactant-polymer formulations (mixtures of xanthan gum or alginate with surfactants) performed best in a laboratory leaf disc bioassay. These formulations significantly improved the efficacy of *S. carpocapsae* against the larvae of the diamondback moth *Plutella xylostella* (L) (Lepidoptera: Plutellidae).

Navaneethan et al. (2010) further confirmed the enhanced effect of the surfactant-polymer formulation for *S. feltiae* against diapausing codling moth larvae (*Cydia pomonella* L., Lepidoptera: Tortricidae). Portman et al. (2016) demonstrated that combining the adjuvants Penterra, Silwet L-77, Sunspray 11N, or Syl-Tac with three species of EPNs (*Heterorhabditis bacteriophora, S. feltiae,* and *S. riobrave*) resulted in higher mortality in wheat stem sawfly (*Cephus cinctus* Norton, Hymenoptera: Cephidae) compared to EPNs applied with water alone in the laboratory assays. Among these adjuvants, Penterra, Silwet L-77, and Syl-Tac are surfactants designed to enhance coverage, wetting, and penetration, while Sunspray 11N is not explicitly classified as a wetting agent. However, paraffinic oils in Sunspray 11N can aid in spreading and adhesion, which are characteristics of some wetting agents.

Mason et al. (1998) observed that the addition of the adjuvants, including surfactants, Crovols, and Triton X-100, significantly increased deposition on Chinese cabbage (*Brassica pekinensis*). Similarly, Beck et al. (2013) found that wetting agents such as Silwet L-77, SBPI, and Addit improved the deposition of *S. carpocapsae* on cauliflower leaves. Kaya et al. (1981) observed positive results using 2% aqueous Stylet or Volck oil adjuvants in EPN applications, although they did not reach statistical significance. Baur et al. (1997) investigated the impact of adjuvants on EPN persistence and efficacy against *P. xylostella*, identifying Rodspray oil as one of the top-performing adjuvants in both laboratory and greenhouse studies. However, a drawback of these adjuvants is that many are laboratory-grade chemicals and thus are either expensive, difficult to prepare, or not readily available.

As the demand for alternative control methods grows, identifying suitable enhancers from readily-available farm or household materials can aid in the utilization of EPNs. In our previous study, a surfactant Southern Ag Surfactant (SAg Surfactant), significantly increased the mortality of the first instars of *Helicoverpa zea* (Boddie) (Lepidoptera: Noctuidae) in corn plants when mixed with *S. carpocapsae* (Zhang et al., 2024). Furthermore, the addition of this surfactant to *S. carpocapsae* applications achieved 92.9% mortality in *C. includens* larvae at an application rate of 40 infective juvenile nematodes (IJs)/cm^2^ in a soybean field. Although the combination of EPNs and this adjuvant showed potential for foliar EPN applications, the effectiveness of this adjuvant compared to its absence has not been tested in soybean plants in the previous study. This study seeks is to evaluate the effects of three easily available adjuvants that could potentially improve the effectiveness of *S. carpocapsae* when used as a foliar application against *C. includens* larvae.

## Materials and Methods

*Insect colony*: Eggs of *C. includens* were purchased from Benzon Research (Carlisle, Pennsylvania). Once hatched, larvae were reared at 27 °C with a 14:10 light-to-dark photoperiod, and 70–80% relative humidity (RH) in environmental chambers (Percival Scientific, Perry, Iowa, USA) using the ARS soybean and wheat germ-based diet developed for *Heliothis virescens* (Blanco et al., 2009a, b).

*EPN colony*: *S. carpocapsae* (ALL strain) was obtained from the USDA-ARS EPN Laboratory in Byron, Georgia, and reared in the last instars of the greater wax moth, *Galleria mellonella* L. (Lepidoptera: Pyralidae), purchased from Josh’s Frogs (Owosso, Michigan). IJs were collected using White traps (Kaya and Stock, 1997). IJs were counted, and suspensions with desired EPN ratios were prepared the day before each experiment by diluting collected IJs with tap water.

*Adjuvants*: The adjuvant treatments used consisted of the following three mixtures: (i) 0.125% dish soap (Dawn Platinum) (hereafter Soap); (ii) 0.125% dish soap combined with 0.25% vegetable oil (Great Value brand, ingredient: soybean oil; hereafter Soap & Oil); and (iii) 0.066% SAg Surfactant (non-ionic, containing active ingredients such as alkyl aryl polyoxyethylene glycol and other ethoxylated derivatives). Adjuvants were premixed with 1 mL of water in 2-mL tubes and then added to the treatment bottles right before application.

*Effect of EPNs with adjuvants on fifth instars of* C. includens *under laboratory conditions*: To assess the impact of adjuvants on the efficacy of EPNs against fifth instars of *C. includens*, four EPN treatments were evaluated at four different exposure times. The four EPN treatments were (i) no adjuvant, (ii) Soap, (iii) Soap & Oil, and (iv) SAg Surfactant. Controls without EPNs were also run for each treatment. Each treatment was replicated three times with 10 insects per replicate, and the entire experiment was conducted twice. Insects were placed individually in 60 × 15 mm Petri dishes lined with a layer of Advantec #2 (thickness 0.26 mm, Advantec MFS, Japan) filter paper. EPN-treated dishes received 1 mL of the EPN suspensions at a rate of 100 IJs/mL, while control dishes received 1 mL of EPN-free solution. There was no standing water on the dishes after application. The dishes were then placed in a dark incubator set at 25 °C, and a water reservoir was placed in the incubator to maintain an RH of 85% or higher for the duration of the experiment. After 1, 4, 8, or 24 hr, the insects were removed from the dishes and placed in diet cups, then returned to the same incubator. Dead larvae were documented daily for 72 hr after application.

*Effect of EPNs with adjuvants on fifth instars of C. includens under greenhouse conditions:* The same adjuvants mentioned above were tested to see if they improved the effect of EPN applications on fifth instars of *C. includens* in a greenhouse, in which we had limited control over temperature or humidity. This experiment included six EPN treatments (combinations of adjuvants and EPNs at various ratios) and four controls. The six EPN treatments were (1) no adjuvants, with 40 IJs/cm^2^ (= 1800 IJs/mL in 30 mL), (2) Soap, with 40 IJs/cm^2^, (3) Soap & Oil, with 40 IJs/cm^2^, (4) SAg Surfactant, with 40 IJs/cm^2^, (5) SAg Surfactant, with 20 IJs/cm^2^ (= 900 IJs/mL in 30 mL), and (6) SAg Surfactant, with 10 IJs/cm^2^ (= 450 IJs/mL in 30 mL). The four EPN-free controls included one with no adjuvant and three with adjuvants, as described in the previous section. Treatments were replicated in triplicate, and the entire experiment was conducted in three trials.

The soybean plants in the greenhouse were grown in 1.9-L pots; most of these had two plants in each pot, but a few had only one plant because the other had died. The plants used were between six and 10 weeks old. Each replicate consisted of seven to 10 plants depending on the sizes of the individual plants; the foliar area was about 1350 cm^2^. Trials were run in the early morning. The temperature and RH at the beginning of treatment, as well as the respective daily minimums and maximums, were recorded using a digital hygrometer and thermometer (AcuRite brand by Chaney Instruments, China). Ten laboratory-reared fifth instars of *C. includens* were used for each replicate, and 30 mL of the desired EPN suspension or control solution were sprayed using a 60-mL pump sprayer in such a way that both the fronts and the backs of the leaves were treated. All replicates received an additional 30 mL of water 2 hr after application.

**Figure 1:**
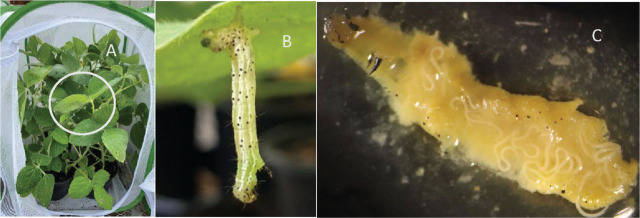
(A) A cage containing soybean plants used for the trials in the greenhouse; the circle over the plants shows where the survival of entomopathogenic nematodes (EPNs) on soybean leaves was examined. (B) A larva of *Chrysodeixis includens* killed by *Steinernema carpocapsae* (ALL strain) within 24 hr in the greenhouse; the color change began from the anus, and EPNs were found after dissection. (C) Adults of *Steinernema carpocapsae* (ALL strain) in the body cavity of a *Chrysodeixis includens* larvae taken from the greenhouse trial.

To prevent loss of test larvae, after application, each replicate’s plant grouping was placed by itself in an insect cage (0.4 x 0.4 x 0.6 m with mesh holes < 0.8 mm) (Insect and Butterfly Habitat Cage Terrarium, Restcloud brand, China) (Fig. 1A). After 24 hr, larvae were removed from the plants and placed individually in diet cups, which were then incubated at room temperature (~22 °C). Dead larvae were documented daily until 72 hr after application.

*Survival of EPNs on the soybean leaves under greenhouse conditions*: The survival of EPNs on the soybean leaves was also examined to determine the effects of these adjuvants under greenhouse conditions. Two leaves of comparable size and age were taken from the centers of the soybean plants (Fig. 1A) in each EPNs treatment replicate to count the viable EPNs present on the leaves. Leaves were examined at 24, 48, 120, and 168 hr after EPNs application for the first trial, but due to hot and dry weather in the greenhouse, this schedule was adjusted to 24, 48, and 96 hr for the second and third trials.

Each leaf was placed individually in a 100 x 15 mm Petri dish for counting EPNs. To both ensure proper wetting and wash off the EPNs, the leaves were soaked in 20 ml of a diluted soap solution for 2 hr, with each leaf turned once halfway through the soaking process. The leaves were then shaken gently to avoid creating excessive bubbles and removed from the Petri dishes. Viable EPNs in the Petri dishes were then counted by placing each dish over a grided dark piece of paper and counting the EPNs under a dissecting microscope. Immobile and non-shrunken IJs were prodded with a fine needle to observe their response to stimuli. Only viable EPNs were counted, as some dead EPNs likely fell off due to the long sampling times of up to 168 hr after spraying (96 hr for the second and third trials). Additionally, it was sometimes difficult to distinguish between dead and shrunken EPNs and hairs that had fallen from soybean leaves. For data analysis, the average number of viable EPNs from the two leaves in each replicate was used to calculate the number of viable EPNs per leaf.

*Data analysis:* Mortality data percentages were normalized using arcsine square root transformation. The number of viable EPNs on soybean leaves was normalized using logarithm transformation before analyzing variance (ANOVA). Treatment means were separated using Tukey HSD in JMP version 15.0.0 (α < 0.05). Untransformed treatment means are presented in the table.

## Results

*Effect of EPNs with adjuvants on fifth instars of C. includens under laboratory conditions*: Because the results at 48 hr were similar to those at 72 hr, only the 24- and 72-hr mortality are reported in Table 1. Overall, the Soap & Oil treatment yielded significantly higher results than the control in five of the sampling time intervals. Soap was significantly higher twice, while SAg Surfactant was significantly higher only once. At 72 hr after application, the Soap & Oil treatment at 1 and 4 hr of exposure, as well as the Soap treatment at 4 hr of exposure, resulted in significantly higher mortality compared to the no-adjuvant treatment. No significant difference in mortality was observed when the exposure time was increased to 8 or 24 hr. However, at 8 hr of exposure, insect mortality 24 hr after application was significantly higher in all the adjuvant treatments compared to the no-adjuvant treatment. Additionally, the Soap & Oil treatment caused significantly higher mortality than no-adjuvant treatment at 4 and 24 hr of exposure.

**Table 1:** Mortality (% ± SEM) of fifth instars of *Chrysodeixis includens* caused by *Steinernema carpocapsae* (ALL strain) with adjuvants at 24 and 72 hr under laboratory conditions (25 °C, 85% RH).

**Exposure (hr)**	**1**		**4**		**8**		**24**	
**Insect dead (hr)**	**24**	**72**	**24**	**72**	**24**	**72**	**24**	**72**
EPN^a^	5.0 ± 2.2 ab	38.3 ± 4.8 b	8.3 ± 4.0 bc	40.0 ± 6.8 b	0.0 ± 0.0 b	63.3 ± 1.2 a	23.3 ± 4.9 b	98.3 ± 1.7 a
EPN+S	8.3 ± 4.0 ab	48.3 ± 6.5 ab	16.7 ± 3.3 b	61.7 ± 7.9 a	15.0 ± 7.2 a	80.0 ± 5.8 a	21.7 ± 5.4 b	98.3 ± 1.7 a
EPN+S+O	10.0 ± 6.3 a	61.7 ± 7.0 a	23.3 ± 4.2 a	66.7 ± 6.1 a	23.3 ± 5.6 a	76.7 ± 6.7 a	51.7 ± 4.0 a	100.0 ± 0 a
EPN+SAg	5.0 ± 3.4 ab	38.3 ± 5.4 b	11.7 ± 6.0 abc	55.0 ± 7.6 ab	10.0 ± 2.6 a	75.0 ± 6.2 a	25.0 ± 3.4 b	98.3 ± 1.7 a
Water	0.0 ± 0.0 b	0.0 ± 0.0 c	0.0 ± 0.0 c	0.0 ± 0.0 c	0.0 ± 0.0 b	0.0 ± 0.0 b	0.0 ± 0.0 c	1.7 ± 1.7 b
Water+S	0.0 ± 0.0 b	0.0 ± 0.0 c	0.0 ± 0.0 c	1.9 ± 1.6 c	0.0 ± 0.0 b	0.0 ± 0.0 b	0.0 ± 0.0 c	0.0 ± 0.0 b
Water+S+O	0.0 ± 0.0 b	1.7 ± 1.7 c	0.0 ± 0.0 c	0.0 ± 0.0 c	0.0 ± 0.0 b	3.5 ± 2.2 b	0.0 ± 0.0 c	0.0 ± 0.0 b
Water+SAg	0.0 ± 0.0 b	1.7 ± 1.7 c	0.0 ± 0.0 c	0.0 ± 0.0 c	0.0 ± 0.0 b	1.9 ± 1.9 b	0.0 ± 0.0 c	0.0 ± 0.0 b

*F*-Value (7, 40)	4.19	58.87	12.08	64.56	11.74	42.17	40.71	457.83
*P*-Value	0.0015	<.0001	<.0001	<.0001	<.0001	<.0001	<.0001	<.0001

Mean values within a column followed by the same letter are not significantly different at *P* > 0.05 (Tukey’s HSD test).

a EPN = Entomopathogenic nematode, S = 0.125% dish soap, S + O = 0.125% dish soap combined with 0.25% vegetable oil, SAg = 0.066% Southern Ag Surfactant.

**Figure 2:**
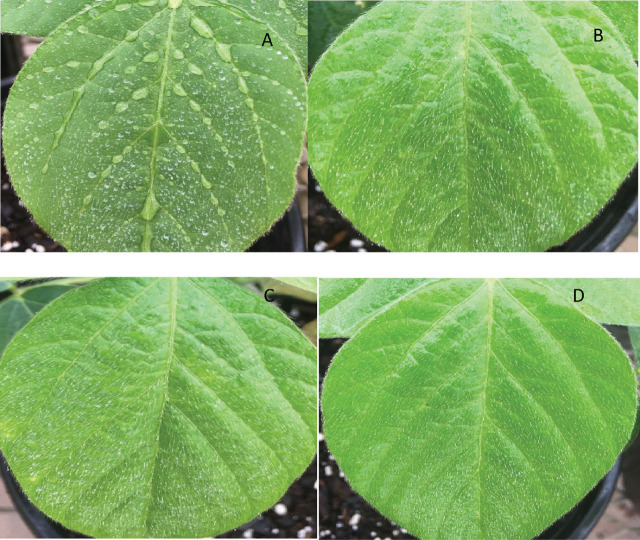
Entomopathogenic nematode (EPN) suspensions after application on soybean leaves. EPN suspension with water only (A) displays numerous water droplets on the leaf. EPN suspensions with dish soap (B), dish soap combined with vegetable oil (C), and Southern Ag Surfactant (D) show the suspensions evenly distributed on the leaves due to the use of adjuvants.

*Effect of EPN with adjuvants on fifth instars of C. includens under greenhouse conditions*: Consistent with the laboratory findings, the insect mortality in the greenhouse at 48 hr was comparable to that at 72 hr, so only the mortality at 24 and 72 hr are reported in Table 2. In the first trial, no dead insects were found in any of the treatments 24 hr after application, but in subsequent trials, when the temperature was higher, dead larvae (Fig. 1B) were found 24 hr after application. Dead larvae were dissected to confirm that their death was caused by EPNs (Fig. 1C). The no-adjuvant treatment showed many water droplets that had formed on the soybean leaves (Fig. 2A). In contrast, the treatments with adjuvants were evenly distributed on foliage (Figs. 2B–D).

**Table 2:** Mortality (% ± SEM) of fifth instars of *Chrysodeixis includens* caused by *Steinernema carpocapsae* (ALL strain) with adjuvants under greenhouse conditions.

**Trial**	**First**	**Second**		**Third**	
Temperature (°C)^a^	19 (10–32)	24 (22–39)		24 (22–38)	
RH (%)^a^	81 (74–90)	81 (44–95)		70 (32–83)	
Plant stage	3^rd^ trifoliolate	Full flowering		Beginning pod	
High (cm)	7.6	40.6		43.2	

Insect dead (hr)	72	24	72	24	72

EPN 40^b^	100.0 ± 0.0 a	19.0 ± 13.2 ab	80.1 ± 8.8 b	7.4 ± 3.7 b	63.0 ± 9.8 b
EPN+S 40	100.0 ± 0.0 a	48.1 ± 19.6 a	100.0 ± 0.0 a	18.5 ± 13.4 ab	77.8 ± 6.4 ab
EPN+S+O 40	100.0 ± 0.0 a	38.4 ± 3.2 a	100.0 ± 0.0 a	48.1 ± 13.4 a	88.9 ± 0.0 a
EPN+SAg 40	100.0 ± 0.0 a	43.5 ± 6.0 a	96.7 ± 3.3 a	11.1 ± 6.4 ab	74.1 ± 7.4 ab
EPN+SAg 20	95.8 ± 7.2 a	14.9 ± 3.7 ab	70.1 ± 4.4 b	3.7 ± 3.7 b	59.3 ± 3.7 bc
EPN+SAg 10	80.0 ± 17.3 b	13.2 ± 8.3 ab	61.4 ± 5.0 b	0.0 ± 0.0 b	32.6 ± 3.4 c
Water	0.0 ± 0.0 c	0.0 ± 0.0 b	0.0 ± 0.0 c	0.0 ± 0.0 b	0.0 ± 0.0 d
Water+S	0.0 ± 0.0 c	0.0 ± 0.0 b	0.0 ± 0.0 c	0.0 ± 0.0 b	0.0 ± 0.0 d
Water+S+O	0.0 ± 0.0 c	0.0 ± 0.0 b	0.0 ± 0.0 c	0.0 ± 0.0 b	0.0 ± 0.0 d
Water+SAg	0.0 ± 0.0 c	0.0 ± 0.0 b	0.0 ± 0.0 c	0.0 ± 0.0 b	0.0 ± 0.0 d
*F*-Value (9, 20)	205.57	7.80	171.92	5.50	83.39
*P*-Value	<.0001	<.0001	<.0001	0.0007	<.0001

Mean values within a column followed by the same letter are not significantly different at *P* > 0.05 (Tukey’s HSD test).

a Numbers outside parentheses were the temperature or RH at the beginning of the application, and numbers inside parentheses were the lowest-highest temperature or RH during the first 24 hr of the trial.

b EPN = entomopathogenic nematode, 40 = 40 IJs/cm^2^, 20 = 20 IJs/cm^2^, and 10 = 10 IJs/cm^2^. S = 0.125% dish soap, S + O = 0.125% dish soap combined with 0.25% vegetable oil, SAg = 0.066% Southern Ag Surfactant.

The results varied by trial. In the first trial (10 to 32 °C, RH 79 to 90%), 72 hr after application, at EPN application rates of 20 and 40 IJ/cm^2^, the mortality among treatments did not differ significantly with or without adjuvants (95.8 to 100.0%), but they were significantly higher than that of SAg Surfactant at the lower rate of 10 IJs/cm^2^ (80.0%). In the second trial (22 to 39 °C, RH 44 to 95%), 24 hr after application, no significant differences were observed among the EPN with and without adjuvant treatments. However, three adjuvant treatments at an EPN application rate of 40 IJs/cm^2^ showed significantly higher efficacy than the controls. Seventy-two hr after application, at an EPN application rate of 40 IJs/cm^2^, all three adjuvant treatments showed significantly higher mortality (96.7 to 100.0%) than the no-adjuvant treatment (80.1%). The mortality caused by the SAg Surfactant treatment at 40 IJs/cm^2^ (96.7%) was significantly higher than that at 20 IJs/cm^2^ (70.1%) and at 10 IJs/cm^2^ (61.4%). In the third trial (22 to 38 °C and RH *32* to 83%), 24 hr after EPNs application at a rate of 40 IJs/cm^2^, only the treatment of Soap & Oil (48.1%) showed significantly higher efficacy than the no-adjuvant treatment (7.4%) and controls. Seventy-two hr after application, at an EPN application rate of 40 IJs/cm^2^, only the treatment of Soap & Oil (88.9%) caused significantly higher mortality than that of no-adjuvant treatment (63.0%). The mortality in the SAg Surfactant treatment at 40 IJs/cm^2^ (74.1%) was not substantially higher than that at 20 IJs/cm^2^ (59.3%), but it was significantly higher than that at 10 IJs/cm^2^ (32.6%). In all trials, EPN treatments caused higher mortality than the no-EPN controls 72 hr after application.

*Survival of EPNs on soybean leaves under greenhouse conditions*: Viable EPNs on soybean leaves were counted 24, 48, 120, and 168 hr after application for the first trial, and 24, 48, and 96 hr for the second and third trials (Table 3). In all three trials, at an EPN rate of 40 IJs/cm^2^, viable EPNs were found in all treatments 24 and 48 hr after application, and in most of the treatments 96 hr (second and third trials) or 120 hr (first trial) after application. However, in the first trial, only a few viable EPNs were found after 168 hr of application. The comparison showed almost no significant difference between adjuvant and no-adjuvant groups at this EPNs rate of 40 IJs/ml (except at 120 hr in the first trial), but Soap & Oil frequently had higher numbers of viable EPNs. In the SAg Surfactant treatments at lower rates of 10 and 20 IJs/cm^2^, viable EPNs were found at 24 hr after application in all three trials, and at 48 hr in the second and third trials. However, no viable EPNs were found 96, 120, or 168 hr after application in any of the trials.

**Table 3:** Number (± SEM) of viable Steinernema carpocapsae (ALL strain) per soybean leaf after application under greenhouse conditions.

**Trail**	**First**				**Second**			**Third**		
Sampling time (hr)	24	48	120	168	24	48	96	24	48	96
Temperature (°C)^a^	10–32	8–32	5–32	5–32	22–39	20–39	20–39	22–38	21–38	20–42
RH (%)^a^	74–90	42–90	42–90	42–90	44–95	44–99	44–99	32–83	26–88	24–94
EPN 40^b^	19.7 ± 3.4 a	3.7 ± 0.9 ab	0.0 ± 0.0 b	0.0 ± 0.0 a	51.7 ± 5.8 ab	10.3 ± 8.1 ab	4.8 ± 1.3 ab	50.0 ± 10.2 a	13.3 ± 7.5 a	0.8 ± 0.4 a
EPN + S 40	20.3 ± 10.9 a	13.5 ± 3.0 a	0.5 ± 0.3 b	0.5 ± 0.3 a	28.8 ± 6.7 ab	12 ± 5.5 ab	4.8 ± 3.1 ab	35.2 ± 25.8 ab	4.7 ± 1.6 ab	0.3 ± 0.2 a
EPN + S+O 40	64.2 ± 36.5 a	15.3 ± 5.7 a	10. 3 ± 4.6 a	0.0 ± 0.0 a	65.2 ± 19.0 a	45.8 ± 5.4 a	9.0 ± 2.8 a	44.8 ± 12.6 a	3.2 ± 1.5 ab	1.2 ± 1.2 a
EPN + SAg 40	9.5 ± 1.8 ab	2.0 ± 0.5 bc	0.5 ± 0.5 b	0.0 ± 0.0 a	33.7 ± 12.9 ab	9.0 ± 3.5 ab	3.7 ± 2.0 ab	21.3 ± 12 ab	5.5 ± 2.4 ab	0 ± 0 a
EPN + SAg 20	8.3 ± 4.4 ab	0.0 ± 0.0 c	0.0 ± 0.0 b	0.0 ± 0.0 a	7.5 ± 4.5 b	4.5 ± 3.0 b	0 .0 ± 0.0 b	6.7 ± 2.4 ab	1.0 ± 0.3 ab	0 ± 0 a
EPN + SAg 10	0.7 ± 0.7 b	0.0 ± 0.0 c	0.0 ± 0.0 b	0.0 ± 0.0 a	4.7 ± 2.4 b	2.8 ± 1.6 b	0.0 ± 0.0 b	1.8 ± 1.4 b	0.0 ± 0.0 b	0 ± 0 a
F-Value (5, 12)	6.59	22.41	16.45	3.32	5.41	4.15	8.40	4.96	3.60	1.14
P-Value	0.0036	<.0001	<.0001	0.0412	0.0078	0.0202	0.0013	0.0108	0.0321	0.3938

Mean values within a column followed by the same letter are not significantly different at *P* > 0.05 (Tukey’s HSD test).

a Lowest-highest temperature or RH.

b 40 = 40 IJs/cm^2^, 20 = 20 IJs/cm^2^, and 10 = 10 IJs/cm^2^.

b EPN = entomopathogenic nematode, S = 0.125% dish soap, S + O = 0.125% dish soap combined with 0.25% vegetable oil, SAg = 0.066% Southern Ag Surfactant.

## Discussion

This study indicates that the three adjuvant treatments — Soap, Soap & Oil, and SAg Surfactant — can enhance the efficacy of *S. carpocapsae* on fifth instars of *C. includens* in the laboratory and greenhouse trials. Overall, Soap & Oil treatment outperformed Soap and SAg Surfactant treatments in terms of the level of mortality achieved. The efficacy of the EPN applications in the trials conducted in different greenhouse conditions (as measured by their mortality) suggests that practical EPN field applications rates can be adjusted and optimized by taking weather conditions into account. In addition, there was some evidence that Soap & Oil may enhance viability, but this was only statistically significant at one sampling time interval. Due to the overlap of some soybean leaves, ensuring that each leaf received approximately the same number of EPNs was challenging. This resulted in large differences among treatment replicates — for example, a high standard error of the mean (SEM) in the mortality in Soap & Oil treatment in the first trial (64.2 ± 36.5) — and this makes it difficult to distinguish among treatments. Nevertheless, the results demonstrated that viable EPNs were still present after 96/168 hr under our experimental conditions.

Previous studies have shown many adjuvants to be non-toxic to IJs (Mason et al., 1998; Schroer et al., 2005a; Beck et al., 2013). Remarkably, Mason et al. (1998) found that while the tested adjuvants were not directly toxic to IJs of two EPN species or to *P. xylostella* larvae, they did affect IJ infectivity in *G. mellonella* larvae (a standard assay for EPN infectivity) by influencing their mortality and infection intensity. Additionally, Krishnayyab and Grewal (2002) reported that exposure to an antibacterial liquid soap (Ajax) at concentrations of 0.12% and 0.23% significantly reduced EPN viability (P < 0.05). Both concentrations, applied alone or in combination with neem oil, resulted in up to 25% mortality of *S. feltiae* IJs after 120 hr of exposure. Similarly, Barbara and Buss (2004) found that 70% of *S. scapterisci* IJs died following exposure to Joy lemon dish detergent at a concentration of 0.4%. Beck et al. (2013) also noted that EPN strains exhibit varying levels of tolerance to different adjuvants. They reported that certain surfactants, including alcohol ethoxylates (specifically Synperonic 91/5, 91/6, 10/6, and Atplus 245), and an alkylpolysaccharide surfactant/humectant (AL-2575), showed strong negative effects on the EPN viability after 3 hr of exposure. While *S. carpocapsae* showed recovery after 15 hr, the impact on *S. feltiae* was persistent, leading to partial or complete loss of viability. In our study, we used the same EPN strain, *S. carpocapsae*, and a soap formulation containing the same chemical group (Alcohols, C9-11, ethoxylated), but at a commercial-grade concentration (1–5%) compared to the technical-grade levels (≥ 90% to ≤ 100%) used by Beck et al. (2013). We also deliberately combined the adjuvants with EPNs only at the onset of the experiment to limit exposure duration.

Soap and SAg Surfactant are wetting agents that reduce water surface tension. A previous study has reported that the use of SAg Surfactant prevented the formation of isolated water droplets that may hinder EPNs from finding hosts (Zhang et al., 2024), and adding SAg Surfactant to *S. carpocapsae* treatments increased the mortality of first instars of *H. zea* in corn fields. This laboratory and greenhouse study supports our previous findings. Additionally, the better performance of the Soap & Oil treatment may be attributed to the role of oil coating the target surface, which reduces the dryness of the leaf surface and provides UV protection (Jones and Burges, 1998). Schroer et al. (2005a) reported that the addition of 0.3% surfactant and either 0.3% xanthan gum or 0.3% potassium alginate to *S. carpocapsae* significantly reduced the lethality time on *P. xylostella* larvae. In our laboratory experiments, compared to insect mortality 24 hr after application, those adjuvant treatments caused significantly higher mortality than the no-adjuvant treatment, suggesting that these adjuvants shortened the time for EPNs to kill *C. includens* larvae.

In our greenhouse experiment, insects were removed 24 hr after treatment. This decision was based on findings from Glazer (1992), who reported that the pathogenicity of *S. carpocapsae* Mexican strain to *Spodoptera littoralis* (F.) (Lepidoptera: Noctuidae) larvae remained almost unaltered when insects were introduced 0, 1, 2 and 4 hr after EPN application on the leaves of 21-days-old bean (*Phaseolus vulgaris*) seedlings in a greenhouse maintained at 65 ± 3% RH and 24 to 26 °C; however, the EPN efficacy dropped dramatically when the insects were introduced for 6 or 8 hr. Schroer et al. (2005b) obtained similar results when using *S. carpocapsae* in a cabbage leaf laboratory bioassay on *P. xylostella* larvae. They observed a rapid decline in mortality when *P. xylostella* larvae were introduced 9 hr after EPN application, and reported that the invasion of *S. carpocapsae* into the *P. xylostella* occurred actively via the anus.

In our greenhouse experiment, dissections of insects frequently revealed EPNs concentrated near the anus (data not shown). Since EPNs rely on a water film for migration, and the water film had dissipated 24 hr after application, even if viable EPNs were present at 24 hr, the EPNs may have lost their ability to actively invade. To simplify our insect assessment and save time, we removed the insects 24 hr after application, allowing for daily observations in cups in the laboratory instead of on plants in the greenhouse.

Low RH is a key limiting factor affecting EPN movement and survival (Glazer, 2002; Navaneethan et al., 2010). That is probably the main reason why the adjuvant’s enhancing effect varied in our greenhouse trials. In the first trial (10 to 32 °C, 74 to 90% RH), there was no difference in mortality among the EPN treatments, whether with or without adjuvants. This lack of difference may be attributed to the EPNs having sufficient time to invade the hosts under those greenhouse conditions. The absence of differences among EPN treatments at rates of 20 and 40 IJs/cm^2^ in that greenhouse trial also suggests that a rate of 40 IJs/cm^2^ may be excessive under the conditions we experienced, and that 20 IJs/cm^2^ might be sufficient. These results suggest that the enhancing effects of the adjuvants may be hidden when the EPN rate is excessive.

In the second trial (22 to 39 °C, 44 to 95% RH), insect mortality from the 40 IJs/cm^2^ treatments was significantly higher in the adjuvant treatments than in the no-adjuvant treatment. Perhaps the time window in which conditions were suitable for EPNs to invade their hosts was shorter than in the first trial. In addition, the mortality (96.7%) at 40 IJs/cm^2^ was significantly higher than that at 20 IJs/cm^2^ (70.1%), suggesting that 40 IJs/cm^2^ might be a more appropriate ratio for these temperature and RH conditions. In the third trial (22 to 38 °C, 32 to 83% RH), only the Soap & Oil treatment (88.9%) was significantly higher than the no-adjuvant treatment (63.0%). It is possible that as the humidity gets lower and lower, the suitable time window for EPNs to invade the hosts gets shorter and shorter. This also suggests that the rate of 40 IJs/cm^2^ might be insufficient here — but under unfavorable environmental conditions, increasing EPNs rates may not be helpful (Navaneethan et al., 2010).

Many factors affect the persistence of EPNs on leaves, such as EPN species, temperature, and RH. Baur (1995) showed that *S. carpocapsae* had a lower LC50 than the other species tested and survived well under desiccating conditions on a leaf surface when used against the diamondback moth, *P. xylostella.* Glazer and Navon (1990) found that survival of *S. feltiae* ALL strain on 10-d-old bean leaves under greenhouse conditions (25 °C, 50–70% RH) was reduced to 20% within 4 hr of exposure and to 0% after 8 hr. In our three greenhouse trials, the longer persistence of EPNs on soybean leaves may be related to the desiccation and UV radiation tolerance of *S. carpocapsae* (Patel *et al.*, 1997; Shapiro-Ilan et al., 2015). Applying EPNs to both the front and the back sides of the leaves may also have increased EPNs persistence.

Our findings support the view that the use of EPNs on foliage is feasible if the optimal conditions are identified (Kaya & Reardon, 1982). Dish soap and vegetable oil are essential materials in every household, and surfactants are also commonly used by farmers. These materials offer convenient access to adjuvants. With the commercialization of EPNs, both large farms and small households can easily purchase them. Many households grow vegetable plants, and the ready availability of these enhancers, combined with commonly-used spraying equipment or bottles, means it is feasible for any household to utilize them. Only one concentration was tested for each adjuvant in this study. Further research is needed to determine if this concentration and formulation are optimal.
